# Correlation Between Advanced Glycation End Products and Ultrasonographic Measurements of Cervico-Facial Skin Tissue

**DOI:** 10.3390/diagnostics16081206

**Published:** 2026-04-17

**Authors:** Anida-Maria Babtan, Claudia Feurdean, Stefan Cristian Vesa, Octavia Sabin, Alexandra-Ioana Roşioară, Sonia Irina Vlaicu, Antonia Eugenia Macarie, Aranka Ilea

**Affiliations:** 1 IIIrd Department—Oral Rehabilitation, Faculty of Dentistry, “Iuliu Haţieganu” University of Medicine and Pharmacy, 400347 Cluj-Napoca, Romania; babtan.anida@umfcluj.ro (A.-M.B.); nicoleta.braitoru@umfcluj.ro (C.F.); aranka.ilea@umfcluj.ro (A.I.); 2 Ist Department—Pharmacology, Toxicology and Clinical Pharmacology, Faculty of Medicine, “Iuliu Haţieganu” University of Medicine and Pharmacy, 400347 Cluj-Napoca, Romania; octaviasabin@gmail.com; 3 IIIrd Department—Hygiene, Department of Community Medicine, “Iuliu Haţieganu” University of Medicine and Pharmacy, 400349 Cluj-Napoca, Romania; alexandra.rosioara@umfcluj.ro; 4 Research Center in Preventive Medicine, Health Promotion and Sustainable Development, “Iuliu Hațieganu” University of Medicine and Pharmacy, 400349 Cluj-Napoca, Romania; 5 IVth Department—Internal Medicine, Faculty of Medicine, “Iuliu Haţieganu” University of Medicine and Pharmacy, 400347 Cluj-Napoca, Romania; vlaicus@yahoo.com; 6 Vth Department—Geriatric-Gerontology, Faculty of Medicine, “Iuliu Haţieganu” University of Medicine and Pharmacy, 400347 Cluj-Napoca, Romania; macarieantonia@yahoo.com

**Keywords:** skin, UV exposure, glycation products, high-frequency ultrasound

## Abstract

**Background/Objectives**: Advanced glycation end products (AGEs) accumulate in long-lived extracellular matrix proteins and have been implicated in skin aging and tissue remodeling, particularly in photo-exposed skin. High-frequency ultrasound (HFU) offers a non-invasive assessment of structural skin parameters that may reflect these changes. This study aimed to explore the associations between serum AGEs and HFU-derived structural parameters of cervico-facial skin, with a focus on UV-exposed dermal tissue. **Methods**: This cross-sectional study included 113 adults recruited in Cluj-Napoca, Romania. Fasting serum samples were analyzed for fructosyl-lysine (FruLys), pyrraline (Pyr), methylglyoxal-derived hydroimidazolone-1 (MG-H1), carboxyethyl-lysine (CEL), carboxymethyl-lysine (CML), arginine (Arg), and lysine (Lys). HFU, using a 22 MHz probe, was performed on the left zygomatic area to assess epidermal depth and density, UV-exposed dermal damage depth and density, dermis depth and density, and subcutaneous tissue depth and density. Associations between serum AGEs and HFU parameters were evaluated using Spearman correlation, with Benjamini–Hochberg false discovery rate (FDR) correction for multiple testing. **Results**: After FDR correction, epidermal depth was inversely correlated with serum CML (r = −0.402, adjusted *p* = 0.018). UV-exposed dermal density was inversely correlated with serum Pyr (r = −0.547, adjusted *p* < 0.019), Arg (r = −0.369, adjusted *p* < 0.019), and Lys (r = −0.270, adjusted *p* < 0.019). Subcutaneous tissue depth was also inversely correlated with serum CML (r = −0.290, adjusted *p* = 0.020). **Conclusions:** The study showed that higher levels of specific serum AGEs were associated with selected HFU-derived structural alterations in cervico-facial skin, particularly in UV-exposed dermal tissue. These exploratory findings support the biological plausibility that systemic glycation may be reflected by non-invasive skin ultrasound parameters.

## 1. Introduction

Health is defined as a state of physical, mental, and social well-being and not merely the absence of disease or infirmity [1]. The global population is continuously aging, and many adults experience age-related pathology and disability. Although the decline in physiological reserves is associated with the aging process, frailty accelerates this decline, and homeostatic mechanisms no longer function within appropriate parameters. Skin aging occurs through two major pathways: intrinsic aging (time-related), which includes collagen fiber breakdown mediated by matrix metalloproteinases (MMPs), inhibition of the transforming growth factor-β (TGF-β) pathway, and the presence of an inflammatory microenvironment [2], and extrinsic aging (photoaging), which is caused by external factors, among which UV radiation exposure is the most important [3]. Skin structure includes keratinocytes, which form the outer protective layer of the skin, as well as collagen, elastin, and proteoglycans, which are key components of the extracellular matrix (ECM) [4]. Skin aging manifests in epidermal layer thinning, flattening of the rete ridges, collagen fiber fragmentation, and decreased collagen synthesis with the onset of an inflammatory microenvironment; these being visible changes with an emotional impact affecting life quality [5].

Skin homeostasis is influenced by photoaging, but also by the presence of glycation (a natural chemical reaction within the human body, involving a non-enzymatic cross-linking of glucose and fructose to proteins, lipids, or nucleic acids [6]. The most representative glycation compounds are advanced glycation end products (AGEs)—the end products of the Maillard reaction, derived from reduced sugars and proteins, lipids or DNA [7]. In metabolic syndrome (MetS), chronic hyperglycemia and oxidative stress accelerate the generation of reactive dicarbonyls (e.g., methylglyoxal—MG-H1) that are converted into AGEs [1,7]. MG-H1 (also found as MGO) is in physiological conditions, a byproduct of the triose phosphate isomerase reaction in glycolysis, eliminated by the glyoxalase system. Hypoxia and inflammation are also determinants in MG-H1 synthesis, because they aggravate glycolysis [5]. In the glyoxalase pathway, the activity of glyoxalase 1 (Glo1) is impaired and will enhance the increased levels of MG-H1, thus explaining its relations to age-related pathologies [8]. AGEs such as carboxymethyl-lysine (CML), carboxyethyl-lysine (CEL), fructosyl-lysine (FruLys), and pyrraline (Pyr), as well as the related amino acids arginine (Arg) and lysine (Lys), are involved in tissue stiffening, inflammation through activation of the AGE receptor (RAGE) and induction of proinflammatory pathways NF κB, metaloproteinases and degradation of the extracellular tissue matrix [9]. There are two AGEs receptors: soluble RAGE (sRAGE) including endogenous secretory RAGE (esRAGE) and metalloproteases cleaved RAGE (cRAGE) [10]. The RAGE transduction caused by IκB kinase (IKKβ) activation promotes phosphorylation and degradation of inhibitor of NF-κB (Iκβ) proteins, thus releasing NF-κB. The transcription of NF-κB enhances the activity of proinflammatory cytokines (IL-1β, IL-6, TNFα) [11]. Impact of the glycation reaction and AGEs on skin structure is as follows: AGEs accumulate in dermal collagen and elastin, causing skin stiffening and loss of elasticity through covalent bonds between collagen/elastin fibers [10], stimulation of inflammation through the RAGE → NF κB → MMP → extracellular matrix activation mechanism, and, most clinically evident, the result of accelerated photo- and chronological aging, manifested by skin yellowing, wrinkles, and dryness. Studies have shown that skin levels of AGEs measured by autofluorescence are correlated with carotid intima-media thickness (a cardiovascular marker) and vascular stiffness [12]. This vessel dysfunction is caused by the cross-linking of elastin and collagen producing stiffness of blood vessels and cardiac fibrosis; low-density lipoproteins (LDLs) also crosslink with AGEs via macrophages which will uptake glycated LDL, enhancing the synthesis of foam cells that induce atherosclerosis [13]. Also, AGEs inhibit nitric oxide (NO) bioavailability and its endothelial synthase, inhibiting antioxidant activity [14]. AGEs measured by autofluorescence in type 1 diabetes are predictive of macro- and microangiopathic complications [15]. Cutaneous AGEs are valuable markers for vascular complications and respond to intensive glycemic management.

Regarding the ultrasound assessment of skin aging, high-frequency ultrasound (HFU), where HFU ≥ 20 MHz, offers precise and non-invasive assessments of structural and inflammatory changes in the skin: it measures the thickness of the epidermis and dermis, echogenicity and the hypoechoic band under the epidermis [16,17]. In MetS, HFU highlights thin epidermis, low cell density, thicker dermis in sun-exposed areas [16]. MetS is considered to be a reservoir for AGEs synthesis, providing substratum for AGEs; both diabetes and dislipidemic status help the formation and accumulation of AGEs, which accumulate in a dose- and time-dependent manner, add up and will deposit in blood vessels and crosslink dermal collagen fibers even after glycemic control is achieved [18].

On the other hand, in autoimmune pathology, in atopic dermatitis, the hypoechoic band under the epidermis is thicker in lesions and present in apparently healthy areas, indicating chronic subclinical inflammation, known as metainflammation [17]. Metainflammation condition is commonly found in MetS patients, in tissues where energy homeostasis is disturbed; its status is upregulated by type 1 pro-inflammatory macrophages (inducing tissue damage), intracellular endoplasmic reticulum dysfunction and constant exposure to pro-inflammatory factors [19].

According to the latest research, HFU has become increasingly used in medical diagnosis, especially in dermatological oncology, but lately also as a diagnostic and monitoring tool in photo-aging, anti-aging moisturizer efficiency treatments, and also plastic surgery procedure outcome monitoring/effects on skin [20,21,22]. HFU has the advantage of highlighting structural skin changes, which reflect the degradation given by the cross-linking of collagen fibers and skin aging through the consequent loss of elasticity; collagen fibers are autofluorescent, derived from their enzymatic and non-enzymatic (glycation) molecular crosslinking, and this fluorescence will be different based on crosslinking density, tissue type, and local factors like pH [23]. AGEs are represented by compounds of exogenous or endogenous origin resulting from the non-enzymatic glycation of protein and carbohydrate groups, and their role in the occurrence and chronicity of systemic inflammation is under continuous research. Moreover, their effect on collagen fibers also has a visible component, which can be quantified by HFU and autofluorescence [20].

Another area explored by HFU is the evaluation of inflammatory skin diseases, such as differentiating between the inflammatory and sclerotic collagen by increasing collagen deposits, which are visible by HFU [24], and examining the skin and determining the degree of tissue aging by monitoring the arrangement of collagen and elastin fibers, the degree of hydration, and the presence and characteristics of the hypoechoic subepidermal band, which tends to change with age [25].

HFU also has applicability in cosmetology pharmacology; in industry and anti-aging therapy (e.g., chemical peeling, platelet-rich-plasma injection), HFU quantifies the effectiveness of treatments through changes in the epidermis and dermal parameters [26]. Thus, HFU offers a non-invasive assessment of structural skin changes and tissue response to interventions in MetS and skin aging. HFU has been used in the evaluation of selected inflammatory skin conditions and changes induced by chronic systemic pathology, such as generating AGEs in the skin structure and collagen aging correlated with predisposing factors—tobacco use, exposure to ultraviolet rays, and consumption of foods from the Western diet category that are cooked at high temperatures with short cooking time (baking, frying, overcooked, pastries)—these being all exogenous sources of AGEs, which will deposit in collagen fibers time- and dose-dependently [27].

The aim of this study was to explore the associations between serum AGEs and HFU-derived structural parameters of cervico-facial skin, with a particular focus on UV-exposed dermal tissue.

## 2. Materials and Methods

This study is analytical, observational, and cross-sectional. Patients (*n* = 113) were recruited from the Departments of Oral Rehabilitation and Oral Prosthetics (Faculty of Dentistry, “Iuliu Hațieganu” University of Medicine and Pharmacy) and from the Regional Diabetes Center in Cluj-Napoca, Romania, between January 2018 and December 2019. Venous blood was selected to assess circulating AGEs levels as potential markers of metabolic status. Given the exploratory design of the study, no formal a priori sample size calculation was performed. The final sample consisted of all eligible participants recruited during the study period.

Inclusion criteria were patients over 18 years of age who required clinical oral examination, with or without dental treatment needs. Exclusion criteria were patients weighing more than 150 kg (which exceeded the assessment capacity of the measuring device) and patients with skin and/or oral mucosa lesions or vascular anomalies. This study was approved by the University Ethics Board, no. 93/8 March 2017. Written informed consent was obtained from all subjects, according to the Declaration of Helsinki of the World Medical Association, revised in 2000, in Edinburgh.

### 2.1. Clinical Evaluation

Patients were asked to report to the Oral Rehabilitation Department at 7:30 a.m. for enrollment, where they were asked to complete written informed consent regarding the procedures that would follow after inclusion in clinical study. Demographic, clinical, and anthropometric measurements were recorded: age, gender, smoking habit, history of diabetes, arterial hypertension and Fitzpatrick skin type. Biological samples were collected under fasting conditions to reduce variability. Venous blood was collected from the upper arm of the included subjects, collected in sterile osmotic tubes, and centrifuged at 4000 rpm/14 min; then, plasma and serum were divided and stored in 100, 200, and 500 µL samples in sterile criotubes. AGEs were quantified as free glycated amino acids and expressed in ng/mL. Fructosyl-lysine (FruLys), methylglyoxal-derived hydroimidazolone-1 (MG-H1), *N*-ε-carboxymethyllysine (CML), *N*-ε-carboxyethyllysine (CEL), and pyrraline (Pyr)—as well as the corresponding unmodified amino acids arginine (Arg) and lysine (Lys)—expressed in µg/mL, were quantified according to the protocol described by Manig et al. [28]. All samples were analyzed by liquid chromatography–tandem mass spectrometry (LC-MS/MS). The performed analysis were single-run type. The biological samples were harvested as the patients were enrolled in the study. For the biochemical analysis, venous blood was harvested in typical tubes for serum, in order to avoid hemolysis. Medical history was reviewed for each patient for any metabolic, autoimmune, degenerative, or oncological disorder. No kidney failure impairment was included in the cross-sectional study. No missing data were present for the variables included in the final analysis.

### 2.2. Ultrasound Evaluation

Potential AGE-related structural changes in collagen-rich tissue were indirectly evaluated [27,29] with a 22 MHz HFU device (DUB cutis, Taberna Pro Medicum, Lüneburg, Germany), with a signal penetration depth of 8 mm and axial resolution of 57 µm at 22 MHz. DUB Cutis HFU device operating parameters are: Scan, RF-Mode, A-Scan, Sum-A, ScanLoop (2000) viewing modes, maximum axial resolution 42 µm at 38 MHz, maximum digitizing depth 16 mm, and 12.8 mm linear (33 µm step width) scan width; for the measurements, DUB cutis automatically records length, area, density, width, depth, ROI (region of interest), rectangular measuring field, and 7 color scales (green shades, yellow, white, black). In this study we used a linear probe (applicator) of 22–28 MHz working in B-Scan (acquisition of sequential ultrasound pulses out in different directions to form 20 image lines). The probe’s maximum penetration/axial resolution is 10 mm/57 µm. HFU acquisition settings were kept constant across all participants, and the transducer was applied with minimal pressure under standardized room conditions. After the acquisition of the images in B mode, the device’s software can automatically evaluate the investigated parameters—density (in percentage of the evaluated area), depth of epidermis and dermis, and echogenity. The color scale helps the software and the practitioner to evaluate the degree of the tissue’s degradation (the more intense the yellow and white area, the greater the degree of collagen fiber cross-linking).

Measurements were performed with the windows covered with curtains (to avoid UV-induced evaluation errors), at an ambient temperature of 25 °C. The ultrasound probe was used as a transmission medium for both the ultrasound gel (applied to the examined surface) and the water introduced into the HFU probe and covered with a thin transparent membrane. Ultrasound images were acquired in B-scan and A-scan viewing modes. The assessment was performed on sun-exposed skin (left zygomatic area) and on non-sun-exposed tissue (non-keratinized mucosa of the lower lip). Before measurements were taken, patients were asked to remove moisturizer/face concealer, if present. The transducer was placed parallel with and without pressure on both the zygomatic and inner lower lip regions after gel application.

**Figure 1:**
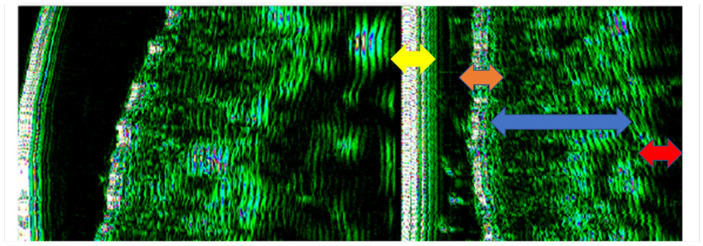
HFU assessment (DUB^®^ cutis, Taberna Pro Medicum) of the lower lip structures, images are exported as pixels in order to highlight tissue density (white and blue—very dense; green—dense; black—very low density or muscular structure). Yellow arrow: transducer membrane; orange arrow: epidermis; blue arrow: dermis; red arrow: hypodermis.

**Figure 2:**
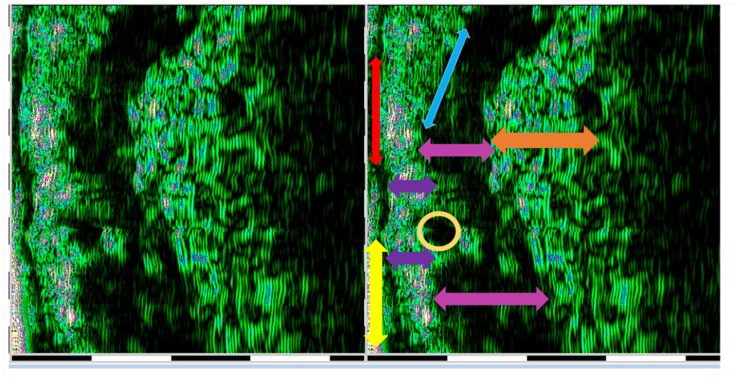
HFU assessment (DUB^®^ cutis, Taberna Pro Medicum) of the lower lip structures. Yellow arrow—superficial layer of non-keratinized squamous epithelium; red arrow—spinous and basal layers of non-keratinized squamous epithelium; purple arrow—papillary and reticular layers of the lamina propria mucosae; blue arrow—limit between lamina propria and reticular layers; orange arrow—tela submucosa; yellow circle—minor salivary gland; pink arrow—orbicularis oris muscle.

Ultrasound measurements were performed by two operators (AMB and SCV). Each assessment was performed three times by each operator. For each examined site, HFU was performed at three different points (mesial, central, and distal). The final value represented the sum of these measurements. When discrepant measurements were observed between operators, the examination was repeated jointly until consensus was reached; however, no predefined numerical discrepancy threshold had been established. Formal reliability assessment was performed for epidermal depth measurements only. The other HFU-derived parameters were obtained using the same standardized acquisition procedure but were not separately subjected to formal reproducibility testing. Intra-rater reliability was assessed for epidermal depth measurements. The correlation coefficient was 0.787, indicating good reliability. Inter-rater reliability was assessed for epidermal depth measurements using Cohen’s kappa coefficient, which revealed a k of 0.881. For the sun-exposed skin of the zygomatic area, including the epidermis, dermis, and subcutaneous tissue (hypodermis), tissue depth (thickness), pixel count (px), and density (automatic) were recorded (Figure 1). The non-sun-exposed tissue examined was the oral mucosa on the inner surface of the lower lip including the non-keratinized epithelium, lamina propria, and submucosa (Figure 2). The following parameters were recorded: depth (thickness) and density.

### 2.3. Statistical Analysis

Statistical analysis was performed using the statistical software MedCalc version 19.2.1 (MedCalc Software Ltd., Ostend, Belgium; https://www.medcalc.org; 2020). Quantitative variables were tested for normality of distribution using the Shapiro–Wilk test and were expressed as median and 25th–75th percentiles. Quantitative data were described using median and 25–75 percentiles. Qualitative data were characterized by frequency and percentage. Correlations between variables were performed using the Spearman coefficient. Partial correlations were performed for the significant AGE–HFU associations, by controlling simultaneously for: age, sex, smoking status, skin phenotype, diabetes, and arterial hypertension. For correlation coefficients, 95% confidence intervals (CI) were estimated using Fisher’s z transformation. Due to the multiple correlation analyses performed, *p*-values were adjusted for multiple comparisons using the Benjamini–Hochberg false discovery rate (FDR) procedure. Statistical significance was interpreted primarily based on FDR-adjusted results.

## 3. Results

Data regarding demographic, HFU evaluation parameters and AGE serum are shown in Table 1.

A weak inverse correlation was observed between epidermal depth and serum CEL (r = −0.248, *p* = 0.008; adjusted *p* = 0.074), and a moderate inverse correlation with serum CML (r = −0.402; *p* < 0.001; adjusted *p* = 0.018) was found (Table 2).

No significant correlation was found between epidermal density and serum AGE (Table 3).

Regarding advanced glycation end products and dermal collagen affected by UV exposure, a weak positive correlation was observed with serum Pyr (r = 0.227, *p* = 0.016); however, after FDR, the correlation did not remain significant (adjusted *p* = 0.112) (Table 4).

Significant correlations were found between dermal collagen density affected by UV exposure as follows: strong inverse correlation with serum Pyr (r = −0.547; *p* < 0.001; adjusted *p* = 0.019), weak inverse correlation with serum CEL (r = −0.241; *p* = 0.011; adjusted *p* = 0.088), moderate inverse correlation with serum Arg (r = −0.369; *p* < 0.001; adjusted *p* = 0.019), and weak inverse correlation with serum Lys (r = −0.270; *p* = 0.004; adjusted *p* = 0.019). After FDR correction, the correlation between serum CEL and UV-exposed dermal density was no longer statistically significant (Table 5).

No significant correlations were found between serum AGEs and dermis depth. (Table 6).

Regarding dermal density and advanced glycation end products in serum, we found a weak direct correlation with serum FruLys (r = 0.203), without statistical significance after FDR (adjusted *p* = 0.192) (Table 7).

Subcutaneous tissue depth was weakly inversely correlated with serum CML (r = −0.290; *p* = 0.002; adjusted *p* = 0.02) (Table 8).

Regarding subcutaneous tissue density we found a weak inverse correlation with serum Lys, without statistical significance after FDR (adjusted *p* = 0.252) (Table 9).

All main significant associations remained statistically significant after adjustment for the available covariates (age, gender, smoking status, diabetes, arterial hypertension, Fitzpatrick skin type) (Table 10).

## 4. Discussion

To our knowledge, the present cross-sectional study is the first to analyze the correlations between serum AGE subtypes (CML, CEL, AGEs modified Arg, Pyr, MG-H1, FruLys, AGEs modified Lys) in serum and skin parameters of the AGEs cross-linking process by HFU (depth and density).

HFU reflects echogenic structural changes potentially associated with collagen remodeling in the dermis and connective tissue. Increased pixelation may reflect reduced dermal elasticity and structural alterations potentially associated with collagen glycation.

Xin et al. evaluated AGEs across all age groups of healthy adults, using fluorescence spectroscopy and laser flowmetry to assess local skin perfusion value and skin elasticity, where their results showed that AGEs are directly correlated with age and dermal decreased vascularization [30]. Also, fibroblast morphology and activity is influenced by high Frulys levels, which activates NFκB—one of the proinflammatory pathways—and contributes to collagen degradation and aging [31].

Clinical signs of collagen degradation (fragility, wrinkles) are driven by high levels of MMPs, which enzymatically degrades and fragmentates skin collagen fibers; from its family, MMP-1 is proven to roughen collagen fiber bundles in dermis, particularly in reticular layers [32]. To this, MMP-2 and MMP-9 are enhanced in activity and lead to the disruption of the extracellular matrix, specifically breaking down laminin, fibronectin and collagen, altering the dermal cellular regeneration, migration, and adhesion. In this manner, the extracellular matrix loses its ability to mechanically sustain fibroblast, resulting in the clinical appearance of aged skin [33].

Our study is the first to demonstrate quantitative correlations between advanced glycation end products (CML, CEL, Arg, Pyr, Lys) in serum and skin parameters of the AGEs cross-linking process by HFU (dermal depth and density). AGEs are deposited in skin collagen and elastin proteins and may lead to structural modifications, especially in skin repeatedly exposed to UV rays [34]. One possible explanation for this finding could lie in the differences in tissue binding properties, biotransformation, and structural significance of the different AGEs. Pyr may have higher stability in relation to long-lived protein structures and may therefore better reflect the cumulative effect of glycation in collagen in chronically UV-exposed skin [35]. Other AGEs such as MG-H1, in contrast, may be less stable, less tissue-retained, or more affected by acute metabolic changes. In our study, serum Pyr was inversely correlated with collagen density in derm that was affected by UV exposure. Kostelc et al. found high values of Pyr at salivary level in patients with gingivitis, related to the inflammatory component of its effects [36]. Pyr has also been linked to skin-related biochemical pathways, although its specific role in dermal structural alterations remains insufficiently understood [37,38].

AGEs are known to have both exogenous and endogenous sources. For example, Arg is a conditionally essential amino acid, its input arriving from protein-rich foods, such as meat, dairy, and nuts, or endogenous synthesis, primarily in the kidney, from dietary glutamate/glutamine, or the third source—liver synthesis included in the urea cycle, from ornithine and carbamoyl phosphate [39].

The changes revealed by HFU are in line with the observations reported by Monnier and Gkogkolou, who showed that the accumulation of AGEs and the fluorescence given by cross-linking are directly correlated with the loss of dermal elasticity and the increase in tissue autofluorescence [40,41]. Our study showed that, with the increase in the degree of damage to collagen by the glycation process in the skin exposed to UV rays, the serum values of glycation products (Arg, Lys and CEL) are lower, which may reflect AGE-related cross-linking of tissue collagen fibers, becoming autofluorescent, which is in agreement with other studies that evaluated the accumulation of AGEs by autofluorescence [17,42,43]. The inverse correlations observed between serum Pyr, Arg, and Lys and UV-exposed dermal density suggest that higher circulating glycation-related burden may be associated with reduced dermal structural density in photo-exposed tissue.

As our study is cross-sectional, the results should not be interpreted as causal or directional. One possible interpretation is that circulating AGEs are associated with structural skin changes, but there are alternative explanations. For example, local AGE accumulation within skin tissue or altered extracellular matrix turnover may influence tissue structure while also contributing to differences in circulating AGE levels. It is also possible that serum AGE levels and HFU-derived skin measurements reflect some processes such as oxidative stress, glycation burden, UV-related remodeling, and metabolic aging [44].

Arg is a non-essential amino acid, with dietary origin and de novo synthesis (intestinal-renal axis) [45]. The literature attests that nitric oxide precursors and arginine methylation (function in ammonia assimilation in the uric cycle and its transformation into urea, creatinine, nitric oxide), together with MG with predominantly dietary origin, are biological markers of reduced glomerular filtration rate and reduced renal glyoxalase-1 (Glo1) function [46,47,48]. Arg plays an important role in multiple roles in cellular metabolism, being implicated in the synthesis of proteins, urea, creatine, γ-aminobutyric acid (GABA), glutamate, and proline, that are important in the human body physiology [48,49]. Skin glycation may also contribute to local structural and phenotypic changes, particularly in UV-exposed skin [50]. Keratinocytes and fibroblasts can participate in AGE-related skin remodeling, while the relatively rapid epidermal turnover may lead to dynamic local accumulation and clearance patterns [51]. These processes may partly explain why the relationship between circulating AGEs and epidermal or dermal HFU parameters is likely to be biologically complex.

The human organism is limited in Arg absorption, the reason for which direct supplementation with arginine is less effective and arginine levels become fluctuating. Arg also is fundamental in skin repair and tissue healing, as Arg activates proliferative and immune defense cellular function due to having a high availability in skin; ultraviolet B (UVB)-exposed human keratinocytes cells release nitrogen oxides, formed from Arg, explaining the inverse correlation with the blood values [52,53]. One possible explanation for the inverse correlation between UV-exposed dermal density and Arg is the effect of chronic UV exposure on collagen fibers, including cross-linking, stiffening, and structural aging, while anabolic and reparative processes may become less active and arginine-related metabolic functions may be altered. We believe that the indirect correlation between UV-exposed dermis and plasma Arg are caused by the influence of UV over collagen fibers—cross-linking, stiffening and aging—whereas anabolic reactions are no more stimulated and Arg synthetic functions are redirected.

CML-related structural alterations may represent a promising area for the study of tissue aging and glycation-related tissue remodeling. The previous literature has reported links between glycation-related tissue changes and inflammatory pathways, although no inflammatory biomarkers were assessed in the present study [54,55,56]. It was also shown that the cross-linking phenomenon can be assessed by HFU and autofluorescence. If confirmed in larger longitudinal studies, HFU may represent a useful non-invasive tool for the assessment of AGE-related tissue alterations. Also, medication and lifestyle-change adjustments could be assessed by skin AGEs quantification, as a non-invasive investigation.

Considering the ultrasound paraclinical examination, the market offers a various range of devices and probes HFU devices are available in both fixed and portable formats [DG-HFUS (Skinscanner) by Dermus, HFUS (DermaScan 20 MHz transducer) by Cortex and DUB Cutis Skin Scanner by Taberna pro Medicum] devices, each one of them with their pros and cons. Visualsonics is a highly sensitive and close-to-histology ultrasound appearance due to its 70 MHz probe, but it has the disadvantage of a non-remote device and a high-price (up to 500–000); in comparison, Skinscanner by Dermus is versatile, also includes a dermatoscope, and has both a laptop- and telephone-linked app [57]. Dermascan by Cortex and DUB Cutis both provide adequate information in order to evaluate real-time skin morphology and the changes within it, as well as the depth of skin lesions.

A weak inverse correlation was observed between serum CML and subcutaneous tissue depth. Similarly, the inverse correlation between serum CML and subcutaneous tissue depth may indicate that higher AGE-related metabolic burden is associated with reduced subcutaneous tissue thickness or structural remodeling. This may reflect the impact of systemic glycation and oxidative stress on adipose tissue structure. AGEs have been shown to induce adipocyte dysfunction and oxidative stress, potentially contributing to adipose tissue remodeling [58]. The strength of the association is modest, and this finding should be interpreted with caution.

Regarding skin health, AGEs, and prevention, community health initiatives should prioritize dietary education on reducing sugar intake, and ultra-processed foods (high in dietary AGEs) to limit the endogenous production of glycation products and reduce glycation-related tissue burden [59]. Strict photoprotection measures should also promote rigorous “UV hygiene” protocols—including the daily use of broad-spectrum sunscreen and physical barriers, while also supporting the potential future use of skin HFU in preventive assessment settings [60]. As our study is an exploratory one, and, given the large number of correlations we performed, the results should be interpreted with caution and will require confirmation from independent cohorts.

### 4.1. Study Limitations

Our study had a cross-sectional design, therefore the results should be interpreted as associations rather than causal relationships. The inclusion criteria were broad to facilitate subjects’ recruitment, but also to have a various sample size of subjects, which might have influenced the correlations. The sample size was adequate to detect moderate associations, but it may have been insufficient to identify weaker associations. No a priori sample size calculation was performed, as the study was designed as an exploratory analysis. Several factors may plausibly influence both circulating AGEs and HFU-derived skin structural parameters, including BMI, UV exposure history and metabolic status. Partial correlations were performed for the main significant associations, but residual confounding cannot be excluded. The reproducibility testing of the HFU examinations was performed only for epidermal depth measurements. Dermal density was measured using the same standardized protocol but was not separately validated for reproducibility. Another limitation is the ultrasonic frequency. The 22 MHz transducer yielded a maximum penetration depth of 8 mm and axial resolution of 57 µm, which permitted examination of the epidermis, dermis, and subcutaneous layers. An increase in frequency could have improved spatial resolution and enhanced sensitivity in detecting structural alterations in the skin. Therefore, the results should be interpreted with caution.

### 4.2. Future Directions

Future studies should confirm these findings in larger, independent cohorts and use longitudinal designs to better clarify the relationship between serum AGEs and HFU-derived skin changes. Also, they need to evaluate the influence of potential confounders, including age, BMI, diabetes, smoking, and UV exposure, and assess the reproducibility of HFU measurements.

## 5. Conclusions

Selected serum AGEs were associated with specific HFU-derived structural skin parameters, particularly in UV-exposed dermal tissue. As these findings are exploratory, they suggest that HFU may capture structural skin changes potentially related to systemic glycation.

## Figures and Tables

**Table 1:** Biochemical and ultrasound assessment in included subjects.

Characteristic		*n* (=113)
**Age**		52 (36; 60)
**Gender**	Male	37 (32.7%)
	Female	76 (67.3%)
**Smoker**		41 (36.3%)
**Diabetes mellitus**		14 (12.4%)
**Arterial hypertension**		35 (31%)
**Skin phenotype (Fitzpatrick)**	II	6 (7.2%)
	III	79 (69.9%)
	IV	27 (23.9%)
**Epidermis depth**		281 (243; 325)
**Epidermis density**		63.31 (47.61; 82.69)
**UV-exposed dermal damage depth**		547 (394.5; 684)
**UV-exposed dermal density**		9.8 (5.4; 17.5)
**Dermis Depth**		1450 (1269.5; 1680)
**Sc tissue depth**		1594 (1094; 2164)
**Sc tissue density**		7.5 (5.2; 12.1)
**Serum FruLys [µg/mL]**		293.5 (205; 380.9)
**Serum Pyr [ng/mL]**		28.4 (25.2; 29)
**Serum MG-H1 [ng/mL]**		33.2 (31.3; 36.3)
**Serum CEL [ng/mL]**		13.3 (12.8; 13.6)
**Serum CML [ng/mL]**		46.9 (44.1; 49)
**Serum Arg [µg/mL]**		5.4 (4.1; 7)
**Serum Lys [µg/mL]**		6.4 (5.7; 7.6)

UV—ultraviolet; Sc—subcutaneous tissue.

**Table 2:** Correlations between serum AGEs and epidermal thickness.

Variable	Epidermis Depth (*n* = 113)		
	R	*p*	95% CI
**serum FruLys [µg/mL]**	0.011	0.904	−0.174 to 0.196
**serum Pyr [ng/mL]**	−0.128	0.177	−0.305 to 0.058
**serum MG-H1 [ng/mL]**	−0.047	0.625	−0.229 to 0.139
**serum CEL [ng/mL]**	−0.248	0.008	−0.414 to −0.066
**serum CML [ng/mL]**	−0.402	<0.001	−0.547 to −0.235
**serum Arg [µg/mL]**	−0.089	0.349	−0.269 to 0.097
**serum Lys [µg/mL]**	−0.138	0.145	−0.315 to 0.048

**Table 3:** Correlations between serum AGEs and epidermal density.

Variable	Epidermis Density (*n* = 113)		
	r	*p*	95% CI
**serum FruLys [µg/mL]**	0.111	0.241	−0.075 to 0.290
**serum Pyr [ng/mL]**	−0.082	0.386	−0.263 to 0.104
**serum MG-H1 [ng/mL]**	−0.003	0.972	−0.188 to 0.182
**serum CEL [ng/mL]**	0.015	0.877	−0.170 to 0.199
**serum CML [ng/mL]**	0.031	0.744	−0.155 to 0.215
**serum Arg [µg/mL]**	0.000	0.999	−0.185 to 0.185
**serum Lys [µg/mL]**	−0.004	0.962	−0.189 to 0.181

**Table 4:** Correlations between serum AGEs and depth of dermal collagen affected by UV exposure.

Variable	UV-Exposed Dermal Damage Depth (*n* = 113)		
	r	*p*	95% CI
**serum FruLys [µg/mL]**	−0.070	0.464	−0.251 to 0.117
**serum Pyr [ng/mL]**	0.227	0.016	0.044 to 0.395
**serum MG-H1 [ng/mL]**	−0.126	0.183	−0.304 to 0.060
**serum CEL [ng/mL]**	0.015	0.877	−0.110 to 0.258
**serum CML [ng/mL]**	−0.115	0.224	−0.294 to 0.071
**serum Arg [µg/mL]**	−0.010	0.912	−0.195 to 0.175
**serum Lys [µg/mL]**	0.071	0.453	−0.115 to 0.253

**Table 5:** Correlations between serum AGEs and the density of the dermis affected by UV exposure.

Variable	UV-Exposed Dermal Density (*n* = 113)		
	r	*p*	95% CI
**serum FruLys [µg/mL]**	0.008	0.937	−0.179 to 0.194
**serum Pyr [ng/mL]**	−0.547	<0.001	−0.666 to −0.402
**serum MG-H1 [ng/mL]**	−0.093	0.332	−0.275 to 0.095
**serum CEL [ng/mL]**	−0.241	0.011	−0.409 to −0.057
**serum CML [ng/mL]**	−0.140	0.143	−0.318 to 0.048
**serum Arg [µg/mL]**	−0.369	<0.001	−0.520 to −0.196
**serum Lys [µg/mL]**	−0.270	0.004	−0.435 to −0.088

**Table 6:** Correlations between serum AGEs and dermis depth.

Variable	Dermis Depth *n* = 113		
	r	*p*	95% CI
**serum FruLys [µg/mL]**	0.073	0.443	−0.113 to 0.254
**serum Pyr [ng/mL]**	−0.017	0.861	−0.201 to 0.169
**serum MG-H1 [ng/mL]**	−0.184	0.052	−0.356 to 0.001
**serum CEL [ng/mL]**	−0.178	0.059	−0.351 to 0.007
**serum CML [ng/mL]**	−0.145	0.126	−0.321 to 0.041
**serum Arg [µg/mL]**	−0.071	0.458	−0.252 to 0.116
**serum Lys [µg/mL]**	−0.044	0.644	−0.227 to 0.142

**Table 7:** Correlations between serum AGEs and dermal density.

Variable	Dermis Density *n* = 113		
	r	*p*	95% CI
**serum FruLys [µg/mL]**	0.203	0.031	0.018 to 0.373
**serum Pyr [ng/mL]**	−0.182	0.054	−0.355 to 0.003
**serum MG-H1 [ng/mL]**	0.067	0.478	−0.119 to 0.249
**serum CEL [ng/mL]**	−0.057	0.551	−0.239 to 0.129
**serum CML [ng/mL]**	0.002	0.979	−0.182 to 0.187
**serum Arg [µg/mL]**	−0.032	0.737	−0.215 to 0.154
**serum Lys [µg/mL]**	−0.052	0.586	−0.234 to 0.134

**Table 8:** Correlations between serum AGE and subcutaneous tissue depth.

Variable	SC Tissue Depth *n* = 113		
	r	*p*	95% CI
**serum FruLys [µg/mL]**	−0.063	0.513	−0.246 to 0.125
**serum Pyr [ng/mL]**	0.013	0.893	−0.174 to 0.199
**serum MG-H1 [ng/mL]**	−0.111	0.248	−0.291 to 0.077
**serum CEL [ng/mL]**	−0.062	0.520	−0.245 to 0.126
**serum CML [ng/mL]**	−0.290	0.002	−0.452 to −0.109
**serum Arg [µg/mL]**	−0.118	0.217	−0.298 to 0.070
**serum Lys [µg/mL]**	0.083	0.388	−0.105 to 0.265

**Table 9:** Correlations between serum AGEs and subcutaneous tissue density.

Variable	SC Tissue Density *n* = 113		
	r	*p*	95% CI
**serum FruLys [µg/mL]**	−0.160	0.093	−0.337 to 0.027
**serum Pyr [ng/mL]**	−0.114	0.233	−0.294 to 0.074
**serum MG-H1 [ng/mL]**	0.087	0.363	−0.101 to 0.269
**serum CEL [ng/mL]**	0.031	0.746	−0.156 to 0.216
**serum CML [ng/mL]**	0.049	0.606	−0.138 to 0.234
**serum Arg [µg/mL]**	−0.075	0.434	−0.258 to 0.113
**serum Lys [µg/mL]**	−0.191	0.045	−0.364 to −0.005

**Table 10:** Partial correlation analyses adjusted for available covariates.

HFU Parameter	AGE	Partial r (Adjusted)	*p*
**Epidermis depth**	**serum** **CML**	−0.361	<0.001
**Epidermis depth**	**serum** **CEL**	−0.200	0.035
**UV-exposed dermal density**	**serum** **Pyr**	−0.524	<0.001
**UV-exposed dermal density**	**serum** **Arg**	−0.374	<0.001
**UV-exposed dermal density**	**serum** **Lys**	−0.260	0.006
**SC depth**	**serum** **CML**	−0.276	0.004

Partial correlations were adjusted simultaneously for age, sex, smoking status, skin phenotype, diabetes, and hypertension.

## Data Availability

Data are available from the corresponding author upon reasonable request.

## Abbreviations

The following abbreviations are used in this manuscript:

AGEs	Advanced glycation end products
Arg	Arginine
CEL	Carboxy-ethyl-lysine
CML	Carboxy-methyl-lysine
CRP	C-reactive protein
Fru-Lys	Fructose-lysine
HDL–C	High-density lipoprotein
HFU	High-frequency ultrasound
IL 6	Interleukin 6
MetS	Metabolic syndrome
MG-H1	Methylglycoxal H1
Pyr	Pyrraline
RAGE	AGE receptor
TNF α	Tumor necrosis factor alpha
Sc	Subcutaneous tissue
UV	Ultraviolet exposure
